# Association of low mixed venous oxygen saturations during early ICU stay with increased 30-day and 1-year mortality after cardiac surgery: a single-center retrospective study

**DOI:** 10.1186/s12871-022-01862-8

**Published:** 2022-10-19

**Authors:** Timo I Kaakinen, Tomi Ikäläinen, Tiina M Erkinaro, Jaana M Karhu, Janne H Liisanantti, Pasi P Ohtonen, Tero I Ala-Kokko

**Affiliations:** 1 Medical Research Center Oulu, Research Group of Surgery, Anesthesiology and Intensive Care Medicine, Oulu University Hospital and University of Oulu, Oulu, Finland; 2 Research Service Unit, Oulu University Hospital, Oulu, Finland

**Keywords:** Pulmonary artery catheter, Cardiac surgery, Goal-directed therapy, Mixed venous oxygen saturation, Postoperative care

## Abstract

**Background:**

Low postoperative mixed venous oxygen saturation (SvO_2_) values have been linked to poor outcomes after cardiac surgery. The present study was designed to assess whether SvO_2_ values of < 60% at intensive care unit (ICU) admission and 4 h after admission are associated with increased mortality after cardiac surgery.

**Methods:**

During the years 2007–2020, 7046 patients (74.4% male; median age, 68 years [interquartile range, 60–74]) underwent cardiac surgery at an academic medical center in Finland. All patients were monitored with a pulmonary artery catheter. SvO_2_ values were obtained at ICU admission and 4 h later. Patients were divided into four groups for analyses: SvO_2_ ≥ 60% at ICU admission and 4 h later; SvO_2_ ≥ 60% at admission but < 60% at 4 h; SvO_2_ < 60% at admission but ≥ 60% at 4 h; and SvO_2_ < 60% at both ICU admission and 4 h later. Kaplan–Meier survival curves, Cox regression models, and receiver operating characteristic curve analysis were used to assess differences among groups in 30-day and 1-year mortality.

**Results:**

In the overall cohort, 52.9% underwent coronary artery bypass grafting (CABG), 29.1% valvular surgery, 12.1% combined CABG and valvular procedures, 3.5% surgery of the ascending aorta or aortic dissection, and 2.4% other cardiac surgery. The 1-year crude mortality was 4.3%. The best outcomes were associated with SvO_2_ ≥ 60% at both ICU admission and 4 h later. Hazard ratios for 1-year mortality were highest among patients with SvO_2_ < 60% at both ICU admission and 4 h later, regardless of surgical subgroup.

**Conclusion:**

SvO_2_ values < 60% at ICU admission and 4 h after admission are associated with increased 30-day and 1-year mortality after cardiac surgery. Goal-directed therapy protocols targeting SvO_2_ ≥ 60% may be beneficial. Prospective studies are needed to confirm these observational findings.

**Supplementary Information:**

The online version contains supplementary material available at 10.1186/s12871-022-01862-8.

## Background

Advanced hemodynamic monitoring to detect disturbed tissue oxygenation, impaired perfusion, and low cardiac output syndrome is crucial during and after cardiac surgery. Pulmonary artery catheter (PAC) has been used in clinical practice since the 1970s [1] and is still considered the gold standard in monitoring cardiac output and pulmonary artery pressures. PAC is the only percutaneous method for obtaining mixed venous oxygen (SvO_2_) samples [2, 3].

PAC-guided goal-directed therapy (GDT) protocols are linked to decreased risk of end-organ dysfunction, such as renal failure, in high-risk surgical patients [4, 5] Tissue hypoperfusion is usually detected by decreased SvO_2_ and/or increased serum lactate levels. SvO_2_ is among the best markers of tissue perfusion [6]. In one randomized controlled trial, GDT targeting SvO_2_ values > 70% up to 8 h after ICU admission following coronary artery bypass grafting (CABG) was associated with reduced complications and shorter hospital stay [7]. However, the level of SvO_2_ to target after cardiac surgery remains unclear. In a retrospective study, SvO_2_ < 60% on ICU admission after CABG was associated with increased 30-day mortality, higher incidence of complications, and prolonged mechanical ventilation and ICU stay [8]. Similarly, in a retrospective analysis of patients undergoing aortic valvular surgery, SvO_2_ < 58% at ICU admission was associated with increased all-cause mortality [9]. Observational data highlight the value of low central venous oxygen saturation (ScvO_2_) in predicting increased mortality after cardiac surgery [10, 11], but ScvO_2_ is not interchangeable with SvO_2_ in absolute values or trends [12].

In our institution, PAC is routinely used in patients undergoing cardiac surgery. SvO_2_ values < 60% are considered low and constitute a threshold for initiating the local GDT protocol for cardiac surgery. We designed the present study to assess the clinical relevance of our practice, specifically to determine whether SvO_2_ values < 60% at ICU admission and 4 h later are associated with increased mortality after cardiac surgery. Our hypothesis was that mortality outcomes at 30 days and 1 year would be associated with SvO_2_ levels in the 4-hour postoperative interval.

## Methods

This single-center retrospective cohort study was conducted in Oulu University Hospital, Oulu, Finland. The data were collected completely from ICU digital medical records. Records for adult patients who had undergone cardiac surgery from January 1, 2007, through December 31, 2020, were reviewed. We chose 2007 as the starting point for data collection because all patient data collected before, during, and after the surgeries, laboratory values, and mortality data were completely digitized at the beginning of 2007.

Each patient was monitored by PAC. Patients were identified using appropriate Acute Physiology and Chronic Health Evaluation (APACHE) II–IV categories during ICU admission and appropriate ICD-10 diagnosis codes. Preoperative risk factors were identified from the Higgins–Cleveland scoring system[13] (years 2007–2012) or the Euroscore [14] (year 2013) or Euroscore II [15] (years 2014–2020) scoring systems. Risk scores were entered by the attending cardiac anesthesiologist before ICU admission. Risk factors identified as common and compatible in each of the three scoring systems were as follows: (1) any type and grade of acute kidney injury, (2) any type of diabetes with insulin therapy, (3) ejection fraction < 35% (or < 30% in Euroscore and Euroscore II), (4) previous cardiac surgery and (5) emergency surgery. These preoperative risk factors were used to adjust the statistical analysis. APACHE II [16] and Sequential Organ Failure Assessment (SOFA) [17] scores on ICU admission also were collected for use in adjusted analyses. The local GDT protocol for cardiac surgery is described in Table S1.

Altogether, 7046 patients were identified. SvO_2_ values at ICU admission and 4 h later were available for 6535 patients. Patients with SvO_2_ > 80% were excluded, as those values are likely to be associated with abnormally low oxygen extraction capacity [18], leaving 6282 patients for the final analysis. Data for these remaining patients were analyzed in four groups based on SvO_2_ values at ICU admission and 4 h later: SvO_2_ ≥ 60% at both time points; SvO_2_ ≥ 60% at ICU admission but < 60% 4 h later; SvO_2_ < 60% at ICU admission but ≥ 60% 4 h later; and SvO_2_ < 60% at both time points. Data on the preoperative risk factors described above were available for 5833 patients, and data on APACHE II and SOFA scores were available for all 6282 patients. Surgical procedures were classified into five categories: ascending aortic repair and aortic dissection, CABG, valvular procedures (single valvular operation), combined procedures (multi-valvular operation or valvular surgery and CABG), and other cardiac surgery (e.g., for various diagnoses such as septal defects, restrictive pericarditis etc.).

### Statistics

We used Kaplan–Meier survival curves with log-rank test values and Cox regression models to assess the differences in 1-year mortality among the groups. Cox regression models were adjusted and analyzed in two ways: with APACHE II score ≥ 25 or < 25 and SOFA score ≥ 8 or < 8 at ICU admission, as well as by sex; or with age, sex, and the preoperative risk factors described above. We give hazard ratios (HRs) with 95% confidence intervals with the Cox models. Receiver operating characteristic (ROC) analysis was conducted to further assess the prognostic value of SvO_2_ values for 30-day mortality. Categorical data are expressed as numbers (n) and percentages (%). Continuous variables are presented as medians with 25th and 75th percentiles. Categorical data were analyzed using Pearson’s chi-square and continuous variables using the non-parametric Kruskal–Wallis H-test.

## Results

Patient characteristics and clinical data are presented in Table 1. Of the 6282 patients, 74.4% were male, and the median age was 68 (interquartile range, 60–74) years. At ICU admission, 3.0% of the patients had an Apache II score ≥ 25, and 19.4% had a SOFA score ≥ 8. The 1-year crude mortality of the whole cohort was 4.3%.

**Table 1:** Patient characteristics and clinical data

Parameter	All (N = 6282)	SvO_2_ ≥ 60% at admission + at 4 h (N = 4585)	SvO_2_ ≥ 60% at admission, < 60% at 4 h (N = 746)	SvO_2_ < 60% at admission, ≥ 60% at 4 h (N = 403)	SvO_2_ < 60% at admission + at 4 h (N = 528)	*P*
Age (years), median (IQR)	68 (60–74)	67 (60–75)	68 (61–74)	70 (63–77)	70 (63–77)	0.052
Male sex, % (n)	74.4 (4657)	74.4 (3408)	79.6 (594)	68.5 (276)	71.8 (379)	< 0.001
BMI (kg/m^2^), median (IQR)	27.3 (24.5–30.4)	27.1 (24.4–30.1)	27.6 (24.7–30.5)	27.7 (25.1–31.0)	28.1 (25.3–31.3)	< 0.001
**Preoperative conditions**, % (n)						
Ejection fraction < 35%^*^	5.0 (292)	4.5 (194)	4.6 (32)	6.0 (22)	9.1 (44)	< 0.001
Insulin-dependent diabetes	15.0 (876)	13.9 (595)	15.1 (105)	19.6 (72)	21.5 (104)	< 0.001
Preoperative acute kidney injury	7.4 (433)	6.9 (275)	10.0 (70)	10.4 (38)	10.3 (50)	< 0.001
**Surgical information**, % (n)						
Previous cardiac surgery	4.9 (288)	4.8 (205)	4.2 (29)	5.7 (21)	6.8 (33)	0.16
Emergency surgery	4.8 (279)	3.5 (148)	5.0 (35)	9.2 (34)	12.8 (62)	< 0.001
CABG	52.8 (3311)	55.0 (2521)	48.1 (359)	54.1 (218)	40.3 (213)	< 0.001
Valvular surgery	29.1 (1825)	30.0 (1372)	29.0 (216)	26.3 (106)	24.8 (131)	< 0.001
Combined procedures	12.1 (756)	9.4 (431)	17.4 (130)	14.1 (57)	26.1 (138)	< 0.001
Ascending aortic repair and aortic dissection	3.5 (217)	3.1 (141)	4.2 (31)	3.7 (15)	5.7 (30)	< 0.001
Other cardiac surgery	2.4 (153)	2.6 (120)	1.3 (31)	1.7 87)	3.0 (16)	< 0.001
**ICU scores**						
APACHE II on admission, median (IQR)	14 (11–17)	13 (11–16)	15 (12–18)	15 (12–18)	16 (13–19)	< 0.001
APACHE II ≥ 25 on admission, % (n)	3.0 (187)	2.0 (91)	3.8 (28)	6.2 (25)	8.1 (43)	< 0.001
SOFA on admission, median (IQR)	6 (5–7)	6 (5–7)	7 (6–8)	6 (5–8)	7 (6–8)	< 0.001
SOFA ≥ 8 on admission, % (n)	19.4 (1216)	15.5 (712)	26.1 (195)	29.0 (117)	36.4 (192)	< 0.001
SOFA max, median (IQR)	6 (5–8)	6 (5–7)	7 (6–9)	7 (6–9)	8 (7–10)	< 0.001
**ICU data**						
Duration of mechanical ventilation (days), median (IQR)	0.24 (0.17–0.42)	0.22 (0.16–0.33)	0.27 (0.17–0.57)	0.29 (0.21–0.71)	0.63 (0.26–1.8)	< 0.001
Renal replacement therapy needed, % (n)	2.4 (149)	1.3 (58)	2.8 (21)	4.7 (19)	9.7 (51)	< 0.001
Max VIS category 0–24 h, % (n)						
0–5	16.7 (1047)	19.2 (285)	11.5 (86)	13.4 (54)	4.7 (528)	< 0.001
5–15	40.0 (2500)	42.9 (1965)	35.9 (268)	34.4 (139)	24.2 (128)	< 0.001
15–30	23.5 (1473)	22.7 (1041)	26.3 (196)	24.8 (100)	25.8 (136)	< 0.001
30–45	6.9 (433)	6.2 (285)	8.3 (62)	7.4 (30)	10.6 (56)	< 0.001
>45	12.9 (809)	9.0 (412)	18.0 (134)	19.9 (80)	34.7 (183)	< 0.001
Max weight gain (kg), median (IQR)	4.5 (2.5–6.6)	4.2 (2.3–6.0)	5.4 (3.4–7.5)	5.0 (3.0–7.8)	6.0 (3.0–8.9)	< 0.001
First 24-h diuresis (mL), median (IQR)	2290 (1865–2820)	2300 (1890–2805)	2300 (1850–2850)	2300 (1850–2900)	2300 (1750–3000)	0.50
First 24-h fluids (mL), median (IQR)	4500 (3800–5400)	4450 (3800–5300)	4600 (3900–5600)	4500 (3700–5300)	4500 (3900–5500)	0.14
Total red blood cell transfusions (units), median (IQR)	2 (1–4)	2 (1–3)	2 (1–4)	2 (1–4)	4 (2–5)	< 0.001
LOS ICU (days), median (IQR)	1 (0.9–2.0)	1 (0.9–1.9)	1.8 (0.9–2.9)	1.7 (0.9–3.6)	2.8 (1.1–4.8)	< 0.001
LOS hospital (days), median (IQR)	8 (7–11)	8 (6–10)	8 (7–12)	8 (7–12)	10 (8–15)	< 0.001
30-d mortality, % (n)	2.1 (132)	1.8 (54)	2.6 (19)	4.5 (18)	7.8 (41)	< 0.001
1-year mortality, % (n)	4.3 (267)	3.0 (137)	5.0 (37)	7.7 (31)	11.8 (62)	< 0.001

*<30% with Euroscore and Euroscore II.

APACHE, Acute Physiology and Chronic Health Evaluation; BMI, body mass index; CABG, coronary artery bypass grafting; ICU, intensive care unit; IQR, interquartile range; LOS, length of stay; SOFA, Sequential Organ Failure Assessment; VIS, vasoactive index score

The groups differed in demographic and clinical data. The group with low SvO_2_ at both time points had the greatest proportions of patients with preoperative ejection fraction < 35% (9.1%), insulin-dependent diabetes (21.5%), emergency procedures (12.8%), combined procedures (26.1%), and surgery for ascending aortic repair or aortic dissection (5.7%). This group also had the longest time on mechanical ventilation (median, 0.63 days), the highest frequency of renal replacement therapy, the highest proportion of patients with maximum vasoactive index scores, highest maximum weight gain (6 kg), and highest amount of red blood cells administered (4 units). They also had the longest stays in the ICU (2.8 days) and hospital (10 days) (Table 1).

Parameters and treatments affecting oxygen supply and demand during the first 4 h after the ICU admission are shown in Table 2. The group with low SvO_2_ at both time points also had the lowest median cardiac index (CI) at ICU admission (2.04 L/min/m^2^). This group and those with low SvO_2_ only at the 4-hour time point both showed increased CI, but values remained lowest in patients with low SvO_2_ at both time points (2.11 L/min/m^2^). Hemoglobin values also were lowest among patients with SvO_2_ < 60% at both time points, as was the P/F ratio at ICU admission and 4 h later, with no increase in the interval. In addition, cumulative fluid volume administered in the ICU within 4 h was the highest in these patients (2000 mL), and this group had the greatest proportion of patients with maximum vasoactive index scores during the 4-hour period.

**Table 2:** Parameters and treatments affecting oxygen supply and demand during the first 4 h after ICU admission

Parameter	All (N = 6282)	SvO_2_ ≥ 60% at admission + at 4 h (N = 4585)	SvO_2_ ≥ 60% at admission, < 60% at 4 h (N = 746)	SvO_2_ < 60% at admission, ≥ 60% at 4 h (N = 403)	SvO_2_ < 60% at admission + at 4 h (N = 528)	*P*
CI on admission (L/min/m^2^)	2.36 (2.03–2.77)	2.43 (2.09–2.83)	2.35 (2.0–2.72)	2.09 (1.8–2.39)	2.04 (1.73–2.36)	< 0.001
CI at 4 h (L/min/m^2^)	2.47 (2.15–2.84)	2.55 (2.23–2.92)	2.28 (2.0–2.59)	2.39 (2.12–2.74)	2.11 (1.87–2.42)	< 0.001
HB on admission (g/L)	105 (96–114)	106 (98–115)	101 (94–110)	101 (91–111)	99 (91–108)	< 0.001
HB at 4 h (g/L)	102 (94–112)	104 (95–114)	98 (90–107)	99 (90–107)	95 (86–102)	< 0.001
Lactate on admission (mmol/L)	1.4 (1.1–1.8)	1.3 (1.1–1.7)	1.4 (1.1–1.8)	1.6 (1.2–2.3)	1.8 (1.4–2.7)	< 0.001
Lactate at 4 h (mmol/L)	1.3 (1.0–1.8)	1.3 (1.0–1.7)	1.5 (1.2–2.0)	1.5 (1.1–2.1)	1.8 (1.4–2.7)	< 0.001
P/F ratio on admission	38 (29–48)	39 (30–49)	36 (28–45)	34 (23–44)	32 (22–43)	< 0.001
P/F ratio at 4 h	40 (30–48)	41 (32–49)	39 (29–48)	37 (27–47)	33 (25–42)	< 0.001
SpO_2_ on admission (%)	97 (95–99)	97 (95–99)	97 (94–98)	96 (93–98)	96 (92–98)	< 0.001
SpO_2_ at 4 h (%)	97 (96–99)	98 (96–99)	97 (96–99)	98 (96–99)	98 (96–99)	0.007
PaO2 on admission (kPa)	15.6 (12.7–19.4)	15.9 (12.9–19.7)	15.3 (12.8–19.0)	14.5 (11.1–17.8)	14.2 (11.5–18.9)	< 0.001
PaO2 at 4 h (kPa)	15.2 (12.7–18.2)	15.5 (12.9–18.4)	14.1 (11.4 (17.1)	15.4 (12.8–19.3)	14.5 (11.9–17.7)	< 0.001
Fluids 0–4 h (mL)	1500 (1050–2200)	1450 (950–2000)	1700 (1300–2400)	1650 (1150–2400)	2000 (1400–2750)	< 0.001
Red blood cell transfusions 0–4 h (units)	1 (1–1)	1 (1–1)	1 (1–1)	1 (1–1)	1 (1–1)	0.67
Max VIS category 0–4 h, % (n)						
0–5	16.7 (528)	18.7 (859)	12.7 (95)	14.6 (59)	5.7 (30)	< 0.001
5–15	29.3 (1838)	30.6 (1402)	26.7 (199)	27.6 (111)	23.9 (126)	< 0.001
15–30	12.8 (804)	12.1 (556)	12.9 (96)	16.4 (66)	16.3 (86)	< 0.001
30–45	2.7 (172)	2.4 (112)	3.1 (23)	3.2 (13)	4.5 (24)	< 0.001
>45	38.4 (2405)	36.1 (1656)	44.6 (333)	38.2 (154)	49.6 (262)	< 0.001

Values are medians (interquartile ranges) unless otherwise specified

CI, cardiac index; HB, hemoglobin; PaO_2_, arterial oxygen partial pressure; P/F, arterial oxygen partial pressure to fractional inspired oxygen ratio; SpO_2_, oxygen saturation; VIS, vasoactive index score

**Fig. 1:**
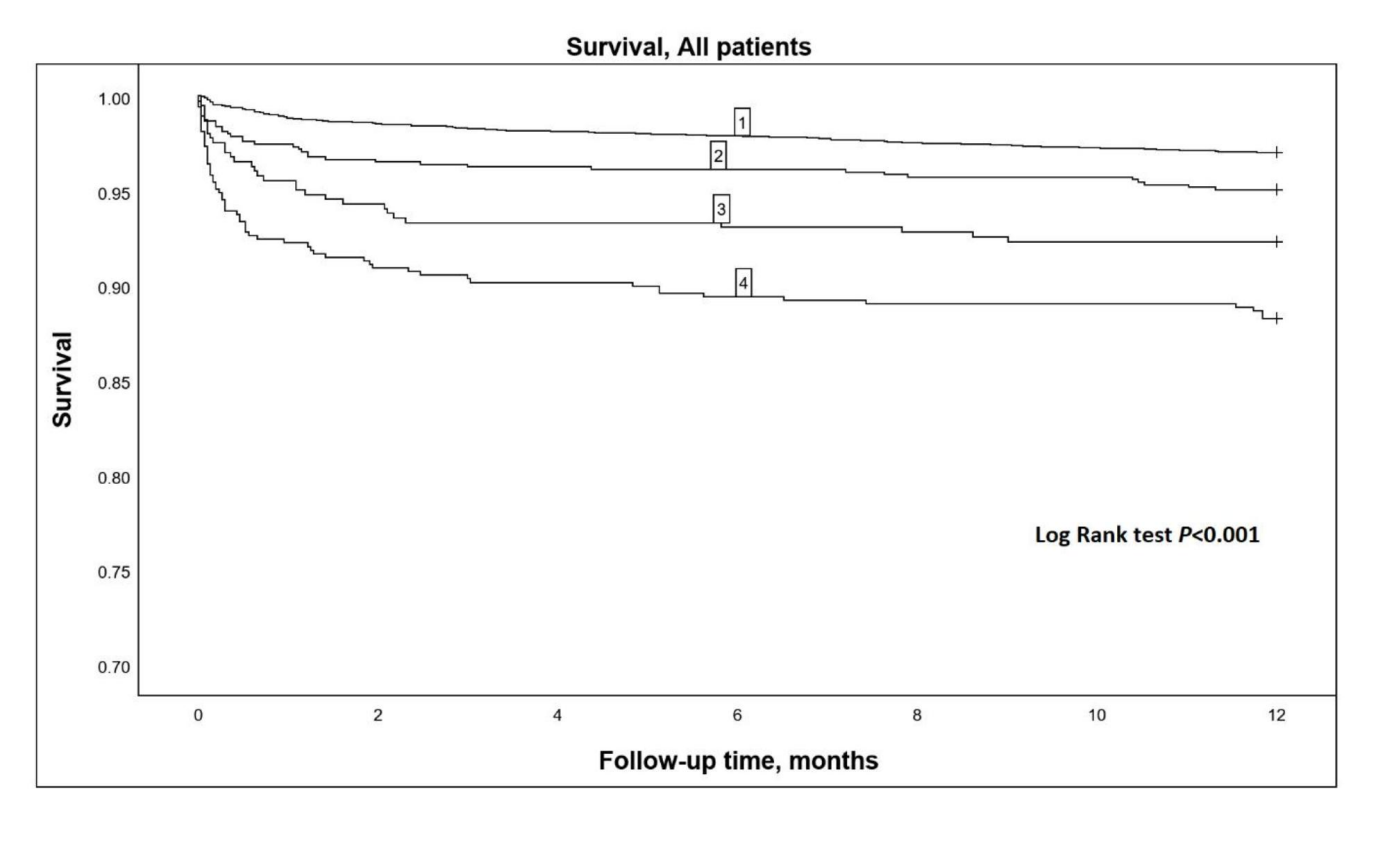
Kaplan–Meier survival curves for the different patient groups (N = 6282). 1, patients with SvO_2_ ≥ 60% at ICU admission and 4 h later (N = 4585); 2, patients with SvO_2_ ≥ 60% at admission but SvO_2_ < 60% 4 h later (N = 746); 3, patients with SvO_2_ < 60% at ICU admission and SvO_2_ ≥ 60% 4 h later (N = 403); and 4, patients with SvO_2_ < 60% at both time points (N = 529)

Cox regression HRs for 1-year mortality are shown in Table 3. The highest HR was observed in the patients with SvO_2_ < 60% at both ICU admission and 4 h later. The results of the Kaplan–Meier analysis are shown in Fig. 1. Having SvO_2_ values ≥ 60% at both ICU admission and 4 h later was associated with the best survival, whereas having SvO_2_ values < 60% at both time points was associated with the poorest survival. Patients with SvO_2_ values < 60% at ICU admission but ≥ 60% 4 h later had better survival than those with low values at both time points. Patients with SvO_2_ values ≥ 60% at ICU admission but < 60% 4 h later had worse survival than patients with consistently high SvO_2_ values but better survival than those with low SvO_2_ at admission and SvO_2_ ≥ 60% at 4 h.

**Table 3:** Crude and adjusted hazard ratios (HRs) and 95% confidence intervals for 1-year mortality. See text for the preoperative risk factors

Group	HR, crude (N = 6282)	*P*	HR, adjusted for sex and Apache II and SOFA scores (N = 6282)	*P*	HR, adjusted for age, sex, and preoperative risk factors (N = 5833)	*P*
**SvO**_**2**_ **> 60% at admission + at 4 h**	1	< 0.001	1	< 0.001	1	< 0.001
**SvO**_**2**_ **> 60% at admission, < 60% at 4 h**	1.69 [1.17–2.42]	0.005	1.46 [1.01–2.1]	0.043	1.70 [1.15–2.51]	0.007
**SvO**_**2**_ **< 60% at admission, > 60% at 4 h**	2.66 [1.8–3.93]	< 0.001	2.05 [1.38–3.04]	< 0.001	1.89 [1.19–2.96]	0.006
**SvO**_**2**_ **< 60% at admission + at 4 h**	4.18 [3.1–5.65]	< 0.001	2.98 [2.18–4.06]	< 0.001	3.19 [2.28–4.45]	< 0.001

The results of the ROC analysis for 30-day mortality are shown in Table 4. SvO_2_ < 60% either at ICU admission or 4 h later predicted 30-day mortality reasonably well, especially after surgery of the ascending aorta and for aortic dissection and combined procedures. Cox regression HRs for each subgroup of surgical procedures are shown in Table 5, and the results of the Kaplan–Meier analysis are given in the supplementary material (Figures S1–S4). In the group with SvO_2_ < 60% at both time points, survival declined significantly during the first 2 months postoperatively, and long-term follow-up survival was shorter than for the other groups. Patients who had undergone “other cardiac surgery” were omitted from the whole subgroup analysis because of low numbers of patients and events. Patients who had undergone ascending aortic repair and aortic dissection were omitted from Cox regression analysis also because of low numbers of patients and events, but ROC analysis and Kaplan–Meier curves are presented. Lower SvO_2_ values at ICU admission and/or 4 h after admission were associated with higher HRs and decreased survival in these subgroups, as well.

**Table 4:** Receiver operating characteristic curve analysis of SvO_2_ for 30-day mortality

Group	SvO_2_ < 60% at ICU admission, AUC (95%CI)	SvO_2_ < 60% 4 h after ICU admission, AUC (95%CI)
All patients	0.71 (0.66–0.76)	0.64 (0.59–0.69)
Ascending aorta and aortic dissection	0.76 (0.66–0.87)	0.74 (0.62–0.86)
CABG	0.66 (0.59–0.74)	0.61 (0.53–0.68)
Valvular surgery	0.68 (0.59–0.78)	0.60 (0.50–0.69)
Combined procedures	0.79 (0.69–0.90)	0.72 (0.61–0.83)

AUC, area under curve; CABG, coronary artery bypass grafting; 95%CI, 95% confidence interval; ICU, intensive care unit; SvO_2_, mixed venous oxygen saturation

**Table 5:** Adjusted hazard ratios (HRs) with 95% confidence intervals for 1-year mortality with different surgical procedures

Surgical subgroup	SvO_2_ status	HR, adjusted for age, sex, and preoperative risk factors (N = 5833)	*P*	HR, adjusted for sex and Apache II and SOFA scores (N = 6282)	*P*
CABG (N = 3311)	≥ 60% at admission + at 4 h	1	0.02	1	< 0.001
	≥ 60% at admission, < 60% at 4 h	1.62 [0.90–2.91]	0.11	1.47 [0.85–2.54]	0.17
	< 60% at admission, ≥ 60% at 4 h	1.79 [0.91–3.53]	0.091	2.42 [1.40–4.19]	0.002
	< 60% at admission + at 4 h	2.28 [1.27–4.09]	0.006	2.52 [1.50–4.24]	0.001
Valvular surgery (N = 1825)	≥ 60% at admission + at 4 h	1	< 0.001	1	0.002
	≥ 60% at admission, < 60% at 4 h	1.35 [0.65–2.8]	0.42	1.09 [0.55–2.16]	0.80
	< 60% at admission, ≥ 60% at 4 h	2.0 [0.84–4.78]	0.12	1.48 [0.69–3.18]	0.32
	< 60% at admission + at 4 h	3.67 [2.01–6.7]	< 0.001	2.97 [1.69–5.22]	< 0.001
Combined procedures (N = 756)	≥ 60% at admission + at 4 h	1	0.003	1	0.002
	≥ 60% at admission, < 60% at 4 h	1.50 [0.47–4.82]	0.50	1.27 [0.40–4.04]	0.69
	< 60% at admission, ≥ 60% at 4 h	1.50 [0.39–5.83]	0.56	2.08 [0.57–7.56]	0.27
	< 60% at admission + at 4 h	4.36 [1.92–9.9]	< 0.001	4.36 [1.96–9.70]	< 0.001

## Discussion

The purpose of this retrospective cohort study was to evaluate whether 30-day and 1-year mortality are associated with SvO_2_ values at ICU admission and 4 h after admission in cardiac surgical patients treated according to the local GDT protocol. The main finding is that SvO_2_ values < 60% at both ICU admission and 4 h later were associated with the highest Cox regression HR values and the highest 1-year mortality.

These results suggest the benefit of monitoring SvO_2_ and using the values as a GDT parameter for guiding therapeutic measures such as fluids and inotropes to improve macrocirculation and support SvO_2_ ≥ 60% after ICU admission. The findings emphasize the importance of intensive hemodynamic monitoring during the first hours after cardiac surgery. Higher mortality in patients with low SvO_2_ on ICU admission, decreasing SvO_2_, or nonresponsive SvO_2_ implies that disturbed tissue oxygenation in the hours immediately after cardiac surgery could significantly affect patient outcomes. The results are in accordance with those of a previous larger study showing an association of SvO_2_ < 60% at ICU admission with worse short- and long-term outcomes and increased perioperative morbidity. The survival benefit with SvO_2_ ≥ 60% at ICU admission has been reported to last up to 2 years (8).

In contrast to our study, previous studies have used only intraoperative [10] or single postoperative SvO_2_ [8, 9] measurement to predict outcomes after cardiac surgery. For each patient, we retrieved two SvO_2_ values flanking the early hours of the postoperative ICU period. Perz et al. explored ScvO_2_ levels during the first hours of the ICU stay after cardiac surgery and found that abnormally low ScvO_2_ normalized within 4–6 h after ICU admission [11]. Although ScvO_2_ values are not interchangeable with SvO_2_ values [12] this result suggests that our time points for SvO_2_ sampling (ICU admission and 4 h later) were suitable for detecting disturbed tissue perfusion in the early postoperative period after cardiac surgery.

In our study, the best outcome was seen with SvO_2_ values maintained above 60% from the time of ICU admission to 4 h later. This group had no overt tissue hypoxemia and had the highest CI levels among all four groups at ICU admission and 4 h after admission. However, even though patients in the other three groups received vasoactive drugs, fluids, and other supportive treatments, decreased survival was noted, and the worst outcome was seen with SvO_2_ remaining < 60%. The pattern of SvO_2_ ≥ 60% at ICU admission but < 60% 4 h later most likely indicated a transient condition related to poor cardiac output or oxygenation, such as postoperative myocardial stunning or hypoxemia. Accordingly, this group showed decreased median CI values from admission to 4 h later. This inference is partly supported by analysis of surgical subgroups showing HRs for these patients that were less significant than in the whole study population, especially for those undergoing valvular and combined procedures. We note that patients with chronic cardiac failure are adapted to lower SvO_2_, which may have had affected our results [19], and measuring preoperative SvO_2_ would have been useful in this context. Additionally, a decreased SvO_2_ may indicate increased oxygen consumption. For example, a small study of 21 cases showed decreasing SvO_2_ in cardiac surgical patients after extubation and that the decline was associated with increased oxygen consumption [20].

SvO_2_ is a marker of whole-body oxygenation, and oxygen use can fail at any level, from the macrocirculatory to microcirculatory to mitochondrial. Patients in the group with low SvO_2_ values at both time points in the current study also represented the most complicated cases, with a third going through combined or aortic surgical procedures with persisting tissue hypoxia. Those with low ICU admission values but higher values at 4 h had existing tissue hypoxia at ICU admission, and despite the SvO_2_ increase during the 4-hour period, mortality remained higher in this group than in patients with sustained SvO_2_ ≥ 60% at both time points. The treatments targeted to increased oxygen delivery may not have yielded increased SvO_2_ because of ongoing tissue hypoxia or mitochondrial dysfunction. In cardiac surgical patients, mitochondrial dysfunction and ongoing oxygen delivery/consumption mismatch could arise from ischemia/reperfusion injury and increased systemic inflammatory response resulting from exposure to cardiopulmonary bypass and surgical tissue trauma [21]. Furthermore, in a study assessing maintenance of SvO_2_ ≥ 60% in high-risk cardiac surgical patients compared with usual care, the protocol group had a higher rate of reintubations and received more fluids, but the duration of norepinephrine infusion was similar between the two groups [22].

SvO_2_ samples are usually obtained using PAC, although other techniques have been described, such as a small catheter inserted into the pulmonary artery during surgery [8, 9]. Data are scarce on the benefits of SvO_2_ or PAC itself in cardiac surgery outcomes. To our knowledge, only one randomized controlled trial has been published that covered postoperative PAC-guided GDT using SvO_2_ as a target parameter in cardiac surgical patients [7]. Studies on the benefit of PAC in cardiac surgery also are scarce, retrospective, or observational, with conflicting results [23–25]. Other major studies of how PAC affects patient outcomes have been conducted in surgical patients, patients with congestive heart failure, or non-cardiac ICU settings [24, 26–30]. However, these studies have not focused on SvO_2_ but rather on filling pressures and cardiac output, and the pre-defined target values are highly variable or nonexistent.

### Strengths

This study has several strengths. To our knowledge, it is the largest study to date showing an association of increased short- and long-term mortality with SvO_2_ < 60% at ICU admission and 4 h later among a cohort of mixed cardiac surgical patients. Our center uses PAC in all cardiac surgical cases, so that health care professionals involved in the care of cardiac surgical patients both intra- and postoperatively are familiar with the use of PAC. Our data collecting system in the ICU is fully automatic, making the data for the present study more reliable because no hand-written information was used. Data on mortality and laboratory values were retrieved from fully digitized hospital records. We had full information on the preoperative risk scores in a clear majority of the patients and could retrieve ICU admission APACHE II and SOFA scores for all patients, enhancing the suitability of our adjusted analyses. In addition to SvO_2_ values, we could present data on postoperative therapy that affects tissue oxygenation, such as administration of vasoactive agents, intravenous fluids, and red blood cell transfusion. Finally, previous studies of the relation between postoperative SvO_2_ values and long-term mortality after cardiac surgery are sparse, and the present study clearly adds to current knowledge.

### Limitations

A main limitation of this work is its single-center retrospective cohort design with all the known related pitfalls, so that the findings are at best hypothesis-generating. Moreover, the group with sustained SvO_2_ < 60% had a significantly higher proportion of patients with preoperative risk factors such as low preoperative ejection fraction, diabetes, and emergency surgery. However, we adjusted the Cox regression analysis with the preoperative risk factors to accommodate this limitation. The study period is long, covering 2007–2020, and during this time, surgical, anesthesia, and perfusion techniques, therapies, and protocols and general care of cardiac surgical patients have evolved. Moreover, the study period incorporates three preoperative cardiac surgical risk scores that we could not fully integrate into our adjustments. However, we focused on SvO_2_ as a risk factor for poor outcome, which is a surrogate of the acute situation, whereas risk scoring systems focus on the preoperative condition. Intraoperative risk factors such as aortic cross-clamping time and perfusion time were not considered in the present study because they are not available in our postoperative ICU data or in the electronic hospital records. However, despite these confounders, we found a survival benefit with SvO_2_ ≥ 60% sustained during the first four postoperative hours after cardiac surgery.

## Conclusion

PAC-obtained SvO_2_ values < 60% at ICU admission and 4 h later are associated with increased 30-day and 1-year mortality after cardiac surgery. GDT protocols targeting maintenance of SvO_2_ ≥ 60% at and following ICU admission with the help of PAC may be beneficial. Prospective randomized controlled studies are needed to confirm these observational findings.

## Electronic supplementary material

Below is the link to the electronic supplementary material.

Supplementary Material 1

Supplementary Material 2

## Data Availability

The datasets used and/or analysed during the current study are not publicly available due to local policy but are available from the corresponding author on reasonable request.

## List of abbreviations

**APACHE**: Acute Physiology and Chronic Health Evaluation.
**CABG**: Coronary artery bypass grafting.
**CI**: Cardiac index.
**GDT**: Goal-directed therapy.
**HR**: Hazard ratio.
**ICU**: Intensive care unit.
**PAC**: Pulmonary artery catheter.
**ROC**: Receiver operating characteristic.
**ScvO**_**2**_: Central venous oxygen saturation.
**SOFA**: Sequential Organ Failure Assessment.
**SvO**_**2**_: Mixed venous oxygen saturation.
**95% CI**: 95% confidence interval.

## References

[1] Swan HJC, Ganz W, Forrester J, Marcus H, Diamond G, Chonette D (1970). Catheterization of the Heart in Man with Use of a Flow-Directed Balloon-Tipped Catheter. N Engl J Med.

[2] Vincent JL. The pulmonary artery catheter. J Clin Monit Comput. 2012. p. 341–5. 10.1007/s10877-012-9389-2 22886686

[3] Szabo C, Betances-Fernandez M, Navas-Blanco JR, Modak RK. PRO: The pulmonary artery catheter has a paramount role in current clinical practice. Ann Card Anaesth. 2021. p. 4–7. 10.4103/aca.ACA_125_19 PMC8081135 33938823

[4] Brienza N, Giglio MT, Marucci M, Fiore T. Does perioperative hemodynamic optimization protect renal function in surgical patients? A meta-analytic study. Crit Care Med. 2009. p. 2079–90. 10.1097/CCM.0b013e3181a00a43 19384211

[5] Svenmarker S, Hannuksela M, Haney M (2018). A retrospective analysis of the mixed venous oxygen saturation as the target for systemic blood flow control during cardiopulmonary bypass. Perfusion.

[6] Kandel G, Aberman A (1983). Mixed Venous Oxygen Saturation: Its Role in the Assessment of the Critically Ill Patient. Arch Intern Med.

[7] Pölönen P, Ruokonen E, Hippeläinen M, Pöyhönen M, Takala J (2000). A prospective, randomized study of goal-oriented hemodynamic therapy in cardiac surgical patients. Anesth Analg.

[8] Holm J, Håkanson E, Vánky F, Svedjeholm R (2011). Mixed venous oxygen saturation predicts short- and long-term outcome after coronary artery bypass grafting surgery: A retrospective cohort analysis. Br J Anaesth.

[9] Holm J, Håkanson RE, Vánky F, Svedjeholm R (2010). Mixed venous oxygen saturation is a prognostic marker after surgery for aortic stenosis. Acta Anaesth Scand.

[10] Miranda C, de Meletti A, Lima JFA, Marchi LHN (2020). Perioperative central venous oxygen saturation and its correlation with mortality during cardiac surgery: an observational prospective study. Braz J Anesthesiol.

[11] Perz S, Uhlig T, Kohl M, Bredle DL, Reinhart K, Bauer M (2011). Low and “supranormal” central venous oxygen saturation and markers of tissue hypoxia in cardiac surgery patients: A prospective observational study. Intensive Care Med.

[12] Lanning KM, Erkinaro TM, Ohtonen PP, Vakkala MA, Liisanantti JH, Ylikauma LA (2021). Accuracy, Precision, and Trending Ability of Perioperative Central Venous Oxygen Saturation Compared to Mixed Venous Oxygen Saturation in Unselected Cardiac Surgical Patients. J Cardiothorac Vasc Anesth.

[13] Higgins TL, Estafanous FG, Loop FD, Beck GJ, Blum JM, Paranandi L (1992). Stratification of Morbidity and Mortality Outcome by Preoperative Risk Factors in Coronary Artery Bypass Patients: A Clinical Severity Score. JAMA.

[14] Nashef SAM, Roques F, Michel P, Gauducheau E, Lemeshow S, Salamon R (1999). European system for cardiac operative risk evaluation (EuroSCORE). Eur J Cardiothorac Surg.

[15] Nashef SAM, Roques F, Sharples LD, Nilsson J, Smith C, Goldstone AR (2012). Euroscore II. Eur J Cardiothorac Surg.

[16] Knaus WA, Draper EA, Wagner DP, Zimmerman JE (1985). APACHE II: A severity of disease classification system. Crit Care Med.

[17] Vincent J-L, Moreno R, Takala J, Willatts S, de Mendonï¿½a A, Bruining H (1996). The SOFA (Sepsis-related Organ Failure Assessment) score to describe organ dysfunction/failure. Intensive Care Med.

[18] Teboul JL, Hamzaoui O, Monnet X. SvO2to monitor resuscitation of septic patients: Let’s just understand the basic physiology. Critical Care. 2011. 10.1186/cc10491 PMC3388677 22078239

[19] Jain A, Shroff SG, Janicki JS, Reddy HK, Weber KT (1991). Relation between mixed venous oxygen saturation and cardiac index; Nonlinearity and normalization for oxygen uptake and hemoglobin. Chest.

[20] Williams J, McLean A, Ahari J, Jose A, Al-Helou G, Ibi I (2020). Decreases in Mixed Venous Blood O2 Saturation in Cardiac Surgery Patients Following Extubation. J Intensive Care Med.

[21] Cherry AD. Mitochondrial Dysfunction in Cardiac Surgery. Anesthesiol Clin. 2019. p. 769–85. 10.1016/j.anclin.2019.08.003 PMC6986803 31677690

[22] Walker LJC, Young PJ (2015). Fluid administration, vasopressor use and patient outcomes in a group of high-risk cardiac surgical patients receiving postoperative goal-directed haemodynamic therapy: A pilot study. Anaesth Intensive Care.

[23] Shaw AD, Mythen MG, Shook D, Hayashida DK, Zhang X, Skaar JR (2018). Pulmonary artery catheter use in adult patients undergoing cardiac surgery: a retrospective, cohort study. Perioper Med (Lond).

[24] Senoner T, Velik-Salchner C, Tauber H. The Pulmonary Artery Catheter in the Perioperative Setting: Should It Still Be Used? Diagnostics (Basel). 2022. p. 177. 10.3390/diagnostics12010177 PMC8774775 35054343

[25] Brown JA, Aranda-Michel E, Kilic A, Serna-Gallegos D, Bianco V, Thoma FW (2021). The impact of pulmonary artery catheter use in cardiac surgery. J Thorac Cardiovasc Surg.

[26] Sandham JD, Hull RD, Brant RF, Knox L, Pineo GF, Doig CJ (2003). A Randomized, Controlled Trial of the Use of Pulmonary-Artery Catheters in High-Risk Surgical Patients. N Engl J Med.

[27] Connors AF, Speroff T, Dawson N, v., Thomas C, Harrell FE, Wagner D (1996). The effectiveness of right heart catheterization in the initial care of critically ill patients. JAMA.

[28] Ivanov RI, Allen J, Sandham JD, Calvin JE. Pulmonary artery catheterization: A narrative and systematic critique of randomized controlled trials and recommendations for the future. New Horiz. 1997. p. 268–76. 9259342

[29] Binanay C, Califf RM, Hasselblad V (2005). Evaluation study of congestive heart failure and pulmonary artery catheterization effectiveness: the ESCAPE trial. JAMA.

[30] Harvey S, Harrison DA, Singer M, Ashcroft J, Jones CM, Elbourne D (2005). Assessment of the clinical effectiveness of pulmonary artery catheters in management of patients in intensive care (PAC-Man): A randomised controlled trial. Lancet.

