# Supplementary material for: Association of low mixed venous oxygen saturations during early ICU stay with increased 30-day and 1-year mortality after cardiac surgery: a single-center retrospective study
Source: BMC Anesthesiol. 2022 Oct 19;22:322. doi: 10.1186/s12871-022-01862-8 (PMC9580133; doi:10.1186/s12871-022-01862-8)
Supplement: Supplementary file 1 — Supplementary Material 1 [file 12871_2022_1862_MOESM1_ESM.pdf]

Digital Supplementary Material for

"Association of Low Mixed Venous Oxygen Saturations During Early ICU Stay With Increased 30-Day And 1-Year Mortality After Cardiac Surgery: A Single-Center Retrospective Study"

Timo I Kaakinen[1] MD, PhD, Tomi Ikäläinen[1], MD, Tiina M Erkinaro[1], MD, PhD, Jaana M Karhu[1], MD, PhD, Janne H Liisanantti[1], MD, PhD, Pasi P Ohtonen[1,2], MSc and Tero I Ala-Kokko[1], MD, PhD

1 Medical Research Center Oulu, Research Group of Surgery, Anesthesiology and Intensive Care Medicine, Oulu University Hospital and University of Oulu, Oulu, Finland.

2 Division of Operative Care, Oulu University Hospital, Oulu, Finland

Corresponding author:

Timo I Kaakinen, MD, PhD

timo.kaakinen@oulu.fi

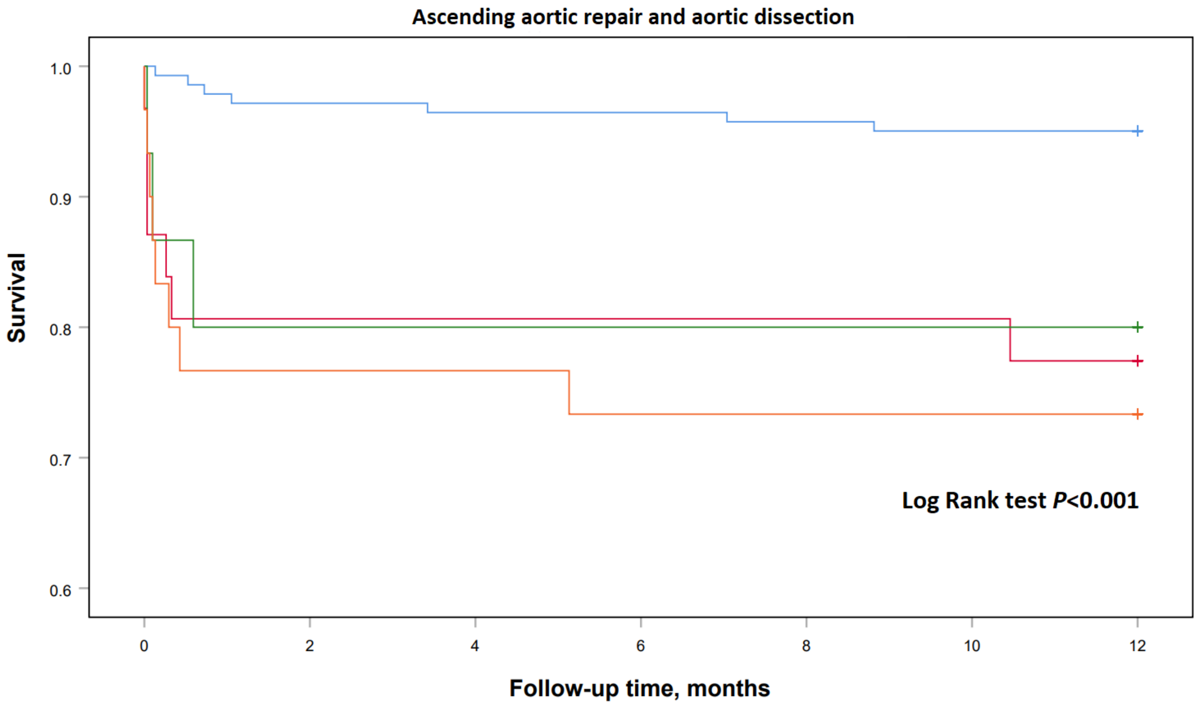

Figure S1. Kaplan–Meier survival curves for patients who underwent ascending aortic repair or aortic dissection (N = 217). Blue, patients with SvO_2 ≥60% at ICU admission and 4 hours later (N = 141); red, patients with SvO_2 ≥60% at admission but SvO_2 <60% 4 hours later (N = 31); green, patients with SvO_2 <60% at ICU admission and SvO_2 ≥60% 4 hours later (N = 15); and orange, patients with SvO_2 <60% at both time points (N = 30).

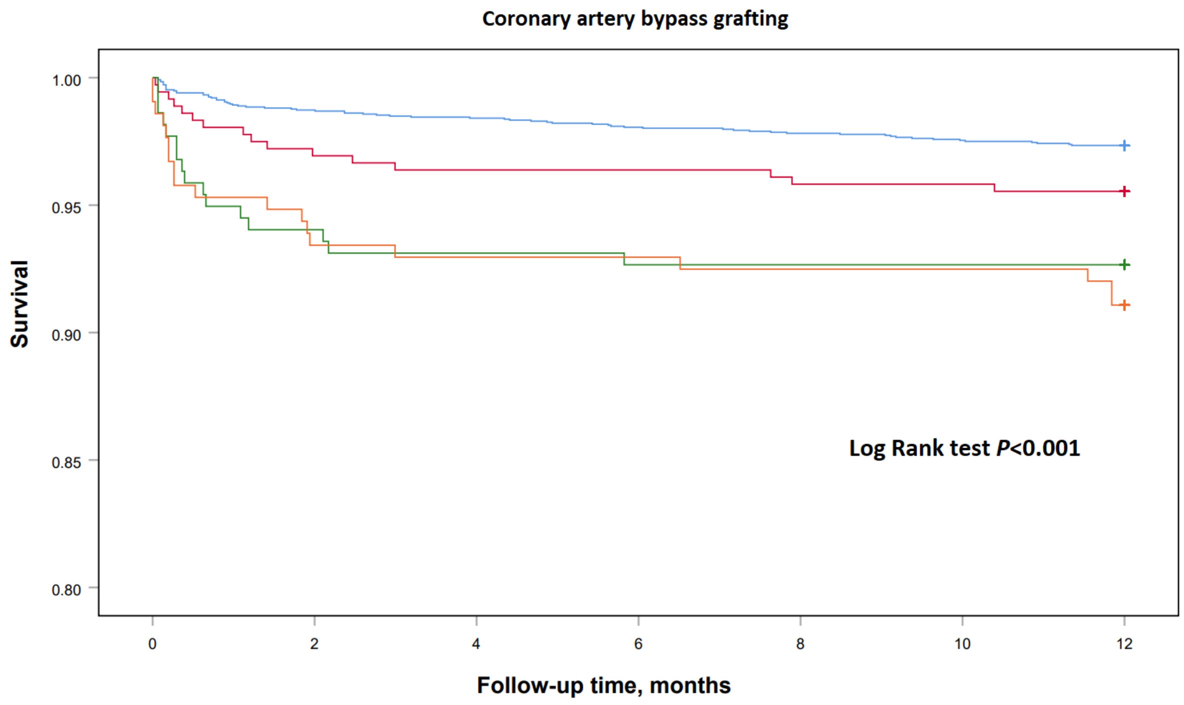

Figure S2. Kaplan–Meier survival curves for patients who underwent coronary artery bypass grafting (N = 3311). Blue, patients with SvO_2 ≥60% at ICU admission and 4 hours later (N = 2521); red, patients with SvO_2 ≥60% at admission but SvO_2 <60% 4 hours later (N = 359); green, patients with SvO_2 <60% at ICU admission and SvO_2 ≥60% 4 hours later (N = 218); and orange, patients with SvO_2 <60% at both time points (N = 213).

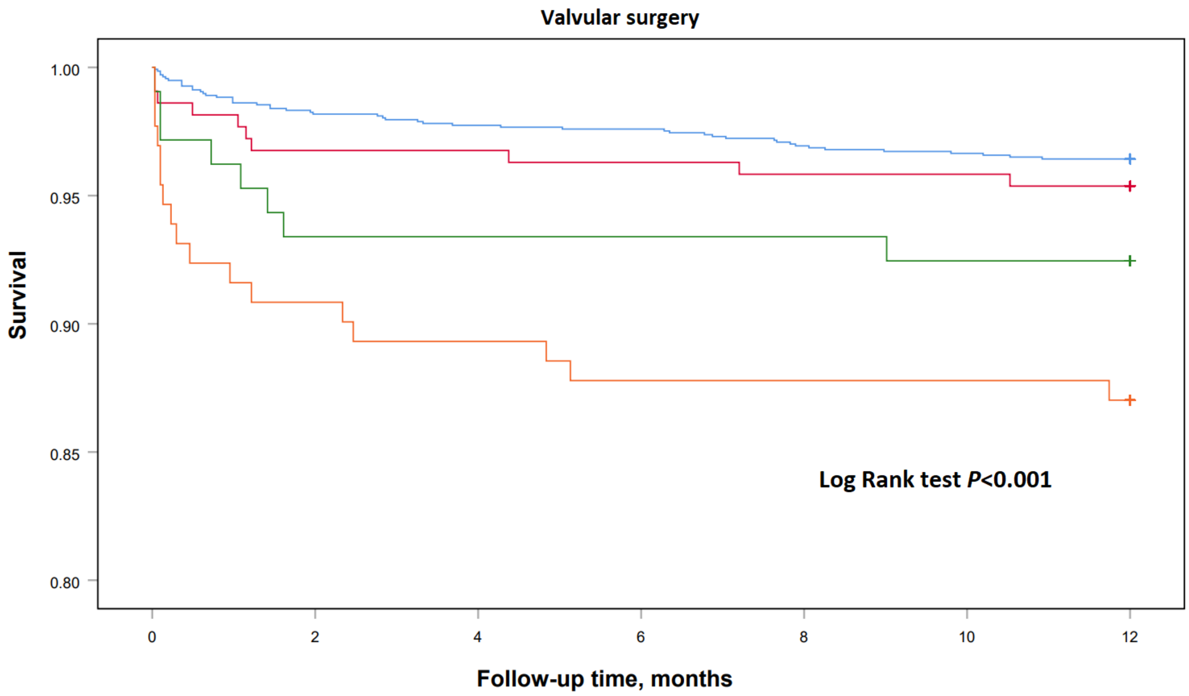

Figure S3. Kaplan–Meier survival curves for patients who underwent valvular surgery (N = 1825). Blue, patients with SvO_2 ≥60% at ICU admission and 4 hours later (N = 1372); red, patients with SvO_2 ≥60% at admission but SvO_2 <60% 4 hours later (N = 216); green, patients with SvO_2 <60% at ICU admission and SvO_2 ≥60% 4 hours later (N = 106); and orange, patients with SvO_2 <60% at both time points (N = 131).

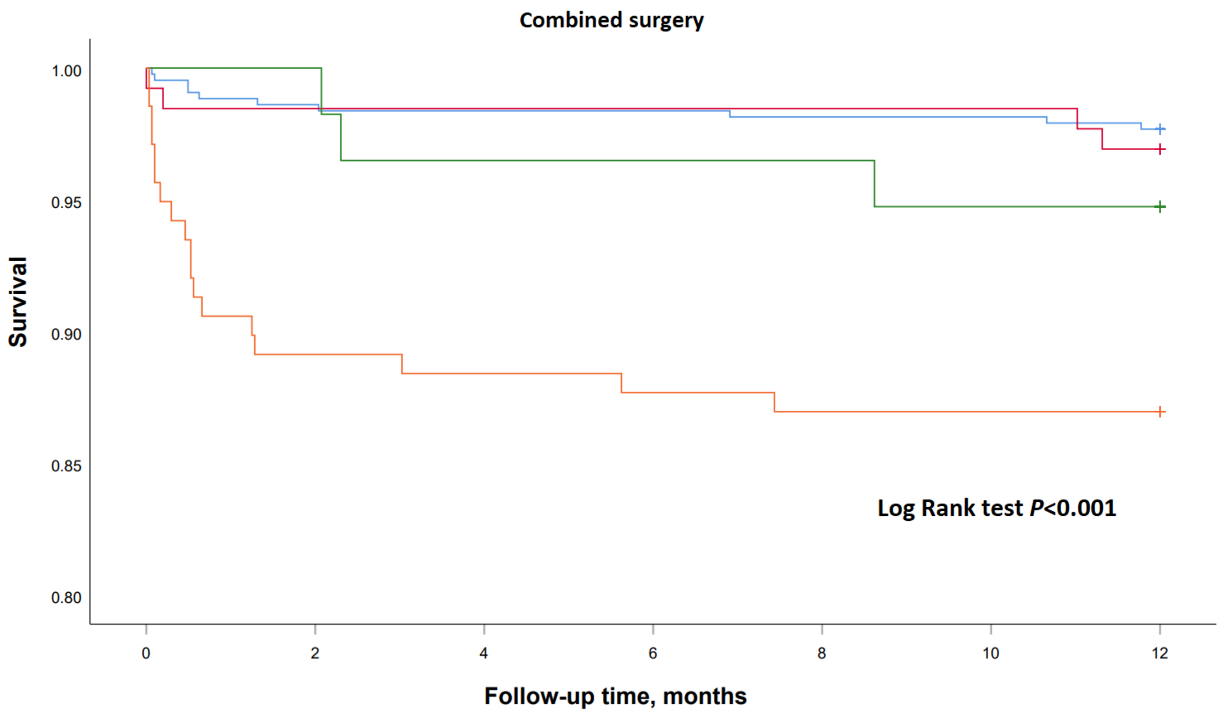

Figure S4. Kaplan–Meier survival curves for patients who underwent combined surgical procedures (N = 756). Blue, patients with SvO_2 ≥60% at ICU admission and 4 hours later (N = 431); red, patients with SvO_2 ≥60% at admission but SvO_2 <60% 4 hours later (N = 130); green, patients with SvO_2 <60% at ICU admission and SvO_2 ≥60% 4 hours later (N = 57); and orange, patients with SvO_2 <60% at both time points (N = 138).
