# Supplementary material for: Association of low mixed venous oxygen saturations during early ICU stay with increased 30-day and 1-year mortality after cardiac surgery: a single-center retrospective study
Source: BMC Anesthesiol. 2022 Oct 19;22:322. doi: 10.1186/s12871-022-01862-8 (PMC9580133; doi:10.1186/s12871-022-01862-8)
Supplement: Supplementary file 2 — Supplementary Material 2 [file 12871_2022_1862_MOESM2_ESM.pdf]

Table S1. Institutional protocol for goal-directed therapy in cardiac surgical patients at the intensive care unit of Oulu University Hospital. In hemodynamic compromise, it is important to rule out myocardial ischemia, cardiac tamponade, ventricular failure, and bleeding. Echocardiography (TTE or TEE) is recommended when the patient suffers from severe hemodynamic disturbance or does not respond to initial therapies. Microcirculatory response is determined with capillary reaction, peripheral temperature, and diuresis (> 0.5 ml/h) as well as SvO_2, acid-base balance and lactate levels, which should be obtained at least every 4 hours.

**SvO_2 < 60% and CI < 2.0 L/min/m^2**	
**signs of bleeding?** – surgical bleeding or coagulation abnormality? TEG/ROTEM recommended before therapy	**signs of ischemia?** consider alerting the cardiologist/cardiac surgeon on call
Hb < 80 g/L? – RBC transfusion	ST-abnormalities on ECG
ACT not in preoperative levels? - protamine	wall motion abnormalities in TTE
INR > 1.5? – FFP transfusion	consider iv-nitroglycerin, check MAP and HR
Thrombocyte count < 100 E9/L? – administer thrombocytes	
Fibrinogen < 1.5-2.0 g/L? – 3-4 g fibrinogen	**signs of hemopericardium/tamponade?** call cardiac surgeon on call/alert the OR
abnormal TEG/ROTEM? – according to findings	pericardial effusion in TTE
probable surgical bleeding – alert the cardiac surgeon and the OR	signs and symptoms suspicious of tamponade
**signs of hypovolemia?** – preload optimization intravenous fluids (balanced crystalloids, albumin)	**signs of arrythmia?**
low CVP, pCWP, pulmonary artery pressures (note that values may not be reliable)	rapid treatment of postoperative atrial fibrillation – cardioversion if hemodynamic compromise
fluid responsiveness improved CI or SV (>10%) with passive leg raising or rapid filling test	temporary pacing in bradycardia
hypovolemia in TTE	
progressive metabolic acidosis	**if bleeding, ischemia, tamponade, arrythmia and hypovolemia are ruled out, consider**
	inotropes – dobutamine, levosimendan, milrinone in left ventricular failure
**hypotension (MAP < 65 mmHg)** – preload optimization first, then norepinephrine	milrinone and inhaled NO in right ventricular failure
vasopressin second line – consider hydrocortisone – remember methylene blue	mechanical support in persistent LCOS – IABP, ECMO

ACT, activated coagulation time; CI, cardiac index; CVP, central venous pressure; ECG, electrocardiogram; ECMO, extracorporeal membrane oxygenation; FFP, fresh frozen plasma; Hb, hemoglobin; HR, heart rate; IABP, intra-aortic balloon pump; INR, international normalized ratio; LCOS, low cardiac output syndrome; MAP, mean arterial pressure; NO, nitric oxide; OR, operation room; pCWP, pulmonary artery wedge pressure; RBC, red blood cells; ROTEM, rotational thromboelastometry; SV, stroke volume; SvO_2, mixed venous oxygen saturation; TEG, thromboelastography; TEE, transesophageal echocardiography; TTE, transthoracic echocardiography.
